# Delineating neuroinflammation, parasite CNS invasion, and blood-brain barrier dysfunction in an experimental murine model of human African trypanosomiasis

**DOI:** 10.1016/j.ymeth.2017.06.015

**Published:** 2017-08-15

**Authors:** Jean Rodgers, Barbara Bradley, Peter G.E. Kennedy

**Affiliations:** a Institute of Biodiversity, Animal Health & Comparative Medicine, College of Medical, Veterinary & Life Sciences, University of Glasgow, Glasgow G61 1QH, UK; b Institute of Infection, Inflammation and Immunity, College of Medical, Veterinary & Life Sciences, University of Glasgow, Glasgow G61 1QH, UK

**Keywords:** Trypanosome, Magnetic resonance imaging, Gadolinium, Blood-brain barrier, Mouse model, Sleeping sickness

## Abstract

Although *Trypanosoma brucei* spp. was first detected by Aldo Castellani in CSF samples taken from sleeping sickness patients over a century ago there is still a great deal of debate surrounding the timing, route and effects of transmigration of the parasite from the blood to the CNS. In this investigation, we have applied contrast-enhance magnetic resonance imaging (MRI) to study the effects of trypanosome infection on the blood-brain barrier (BBB) in the well-established GVR35 mouse model of sleeping sickness. In addition, we have measured the trypanosome load present in the brain using quantitative Taqman PCR and assessed the severity of the neuroinflammatory reaction at specific time points over the course of the infection.

Contrast enhanced–MRI detected a significant degree of BBB impairment in mice at 14 days following trypanosome infection, which increased in a step-wise fashion as the disease progressed. Parasite DNA was present in the brain tissue on day 7 after infection. This increased significantly in quantity by day 14 post-infection and continued to rise as the infection advanced. A progressive increase in neuroinflammation was detected following trypanosome infection, reaching a significant level of severity on day 14 post-infection and rising further at later time-points. In this model stage-2 disease presents at 21 days post-infection.

The combination of the three methodologies indicates that changes in the CNS become apparent prior to the onset of established stage-2 disease. This could in part account for the difficulties associated with defining specific criteria to distinguish stage-1 and stage-2 infections and highlights the need for improved staging diagnostics.

## Highlights

• CE-MRI detected significant BBB impairment in mice at 14 days following trypanosome infection.
• BBB dysfunction increased in a step-wise fashion as the disease progressed.
• Parasite DNA was present in the brain tissue on day 7 after infection.
• Parasite CNS load continued to rise as the infection advanced.
• A neuroinflammatory reaction developed quickly following infection.
• The severity of the neuropathological reaction increased at later time-points.
• Changes in the CNS are apparent prior to the onset of established stage-2 disease.

## 1 Introduction

Human African trypanosomiasis (HAT), also known as sleeping sickness, is caused by infection with the parasitic protozoans *Trypanosoma brucei gambiense (T.b.gambiense)* or *Trypanosoma brucei rhodesiense (T.b.rhodesiense)* and is spread by the bite of the tsetse fly insect vector [1]*.* The disease is usually fatal if not diagnosed and treated with appropriate chemotherapy. *T.b.gambiense* is by far the more prevalent of the two infections and is responsible for approximately 97% of all reported cases with *T.b.rhodesiense* accounting for the remaining 3%. The parasites are restricted to sub-Saharan Africa where approximately 70 million people are at risk of infection. However, following the application of effective control and surveillance strategies the number of reported new cases fell to less than 10,000 in 2009 and has continued to decline with less than 3000 new cases recorded in 2015 [2]. These figures suggest that elimination of the disease, defined as less than one case per 10,000 population in at least 90% of endemic areas, by 2020 is an achievable target. Nevertheless, the reported case number likely depicts a significant under representation of the scale of the problem and WHO estimate that the actual case number is closer to 20,000 [1]. Re-emergence of the disease to epidemic levels has occurred historically [3] and this recurrence emphasises the necessity to sustain current control strategies.

Following infection the disease progresses through two stages. During stage-1 the parasites proliferate in the blood, lymphatic system and peripheral organs. However, the most serious form of HAT, stage-2, occurs when the trypanosomes circumvent the blood-brain barrier (BBB) to enter and establish within the CNS. Gambiense infections are associated with a chronic progressive course and can take months to years before stage-2 disease is reached, while *rhodesiense* infections are more acute with parasites entering the CNS within a matter of weeks [4]. The progression of the infection to the CNS-stage is associated with the development of a neuroinflammatory reaction described in only a limited number of human cases [5], [6]. This neuroinflammatory response to trypanosome infection has been mirrored in both rodent and primate models of the human disease and is characterised by inflammatory cells, including lymphocytes, macrophages and plasma cells, infiltrating the meninges and choroid, followed by inflammation of the parenchymal vessels and lastly the development of encephalitis. Astrocyte and microglial cell activation accompany this response although little neuronal damage or demyelination occur until the terminal stages of the disease are reached [5], [7], [8], [9].

The precise microenvironment required within the brain to conserve optimal function is maintained by the presence of specialised barriers situated between the neural tissue and the circulating blood [10]. These barriers protect the brain from the vast majority of toxins and pathogens as well as regulate the exchange of nutrients, metabolites, molecules and ions between the brain parenchyma and the blood by means of specific transporters and ion channels [11]. The most extensive barrier type is found between the blood and the brain parenchyma and is formed through a complex functional interplay between brain microvascular endothelial cells, which are bound together by sophisticated ‘tight junctions’, pericytes, astrocytes, neurons and microglial cells. Together these cells constitute the neurovascular units that comprise the ‘classical’ parenchymal BBB [11]. There are additional barriers in the choroid plexus separating the blood from the ventricular CSF, and between the blood and subarachnoid CSF. These barriers have endothelial cells joined by tight junctions but lack the other cellular components of the parenchymal BBB [12].

Numerous neurological conditions, of both infectious and non-infectious aetiology, can initiate various degrees of BBB impairment [13], [14], [15]. However, the impact of trypanosome infection on BBB function remains controversial [16], [17], [18], [19]. Rhodamine dye, injected into the jugular vein of rats during the advanced stages of *T.b.brucei* infection, has been found permeating the brain cortical white and grey matter, indicating the presence of BBB dysfunction in these animals [17]. In a similar rat model, Mulenga et al. [16] detected increasing numbers of parasites in the brain parenchyma as the infection progressed, though no changes were seen in the staining patterns of the tight junction proteins occludin and zonula occludens 1, or the penetration of fibrinogen or IgG. In this instance, the findings suggest that trypanosome infection and transmigration into the CNS does not result in loss of BBB integrity. The application of an *in vitro* BBB model utilising human brain microvascular endothelial cells has provided further evidence suggesting that trypanosomes do not cause lasting damage to the BBB [19]. This study showed that *T.b.rhodesiense* induced only a transient reduction in transendothelial electrical resistance (TEER), which was most pronounced around 3 h following introduction of the parasites [19].

More recent studies, employing contrast-enhanced magnetic resonance imaging (CE-MRI) to investigate BBB function in a murine model of HAT, demonstrated significant and widespread BBB dysfunction during CNS-stage disease [20]. Furthermore, the barrier impairment was present in animals displaying only mild to moderate neuroinflammatory changes in the brain; typically comprised of inflammatory cells in the meninges and the development of perivascular cuffs around some of the blood vessels. In the investigation presented here we have extended these findings to ascertain when BBB impairment becomes apparent following *T.b.brucei* infection and measured the severity of the dysfunction. In addition, the degree of neuroinflammation and the trypanosome burden in the brain has been determined during the progression of the disease.

## 2 Materials and methods

### 2.1 Animals and infections

All animal experiments were approved by the University of Glasgow Ethical Review Committee and performed in accordance with the ARRIVE guidelines, UK Animals (Scientific Procedures) Act, 1986 and EU directive 2010/63/EU.

The well-established *Trypanosoma brucei* (*T.b.*) *brucei* GVR35 mouse model of human African trypanosomiasis was used throughout this study. Briefly, 45 female CD-1 mice were infected by intraperitoneal injection of 2 × 10^4^ parasites in 100 μL phosphate buffered saline glucose (PBS-G). The animals were divided into three cohorts and assigned to study; the neuroinflammatory reaction (n = 20), the trypanosome load (n = 20) or BBB function (n = 5). Each cohort was further divided into sub-groups (n = 5) to investigate disease progression at 7, 14, 21 and 28 days post-infection. Only one group was allocated to MRI as serial scans were performed on individual animals at each time point. Uninfected animals were included with the neuroinflammation (n = 4) and MRI (n = 3) studies to act as normal controls.

### 2.2 Histopathology

The severity of the neuroinflammatory reaction was assessed in groups of mice sacrificed at each time-point. At sacrifice the animals were perfused transcardially with approximately 120 mL sterile saline. The brains were then excised, fixed in 4% neutral buffered formalin, and paraffin-wax processed. Coronal sections, taken through the hippocampal brain region, were then prepared and stained with haematoxylin and eosin. The stained sections were assessed in a blinded fashion and the severity of the neuroinflammatory reaction graded using a previously described grading scale [21]. Briefly, a score of 0 describes a normal brain, grade 1 describes sections where a mild meningitis is present while grade 2 shows a moderate meningitis with perivascular cuffing of some vessels. Grade 3 is characterised by more severe meningitis and perivascular cuffing with a few inflammatory cells infiltrating the neuropil, and grade 4 describes a severe meningoencephalitis with inflammatory cells throughout the brain parenchyma.

### 2.3 Quantitative PCR

Trypanosome load in the brain was determined using Taqman real-time PCR as described previously [22], [23]. Mice were euthanased at 7, 14, 21 and 28 days -post-infection and perfused transcardially with 120 mL of sterile saline to remove peripheral blood from the CNS. The brains were then excised, immediately placed in dry ice and stored at −70 °C until required. DNA was prepared from a 25 mg sample of whole brain homogenate (DNeasy Tissue kit; Qiagen) and Taqman real-time PCR performed [22], [23]. Briefly, Taqman PCR was carried out in a 25 μL reaction volume comprising 1 x Taqman Brilliant II master mix (Agilent), 0.05 pmol/μL forward primer (CCAACCGTGTGTTTCCTCCT), 0.05 pmol/μL reverse primer (GAAAAGGTGTCAAACTACTGCCG), 0.1 pmol/μL probe (FAM-CTTGTCTTCTCCTTTTTTGTCTCTTTCCCCCT-TAMRA) (Eurofins MWG Operon) and 100 ng template DNA. The primers and probe were specifically designed to detect the trypanosome *Pfr*2 gene. A standard curve, constructed using a serial dilution of pCR®2.1 vector containing 1 × 10^6^–1 × 10^1^ copies the cloned *Pfr*2 target sequence (Eurofins MWG Operon), was include in each PCR plate. The amplification was performed using a MxPro 3005 (Agilent) with a thermal profile of 95 °C for 10 min followed by 45 cycles of 95 °C for 15 s, 60 °C for 1 min and 72 °C for 1 s. The trypanosome load within the brain samples was extrapolated from the standard curve using the MxPro qPCR software (Agilent).

### 2.4 Contrast enhanced magnetic resonance imaging

**Fig. 1:**
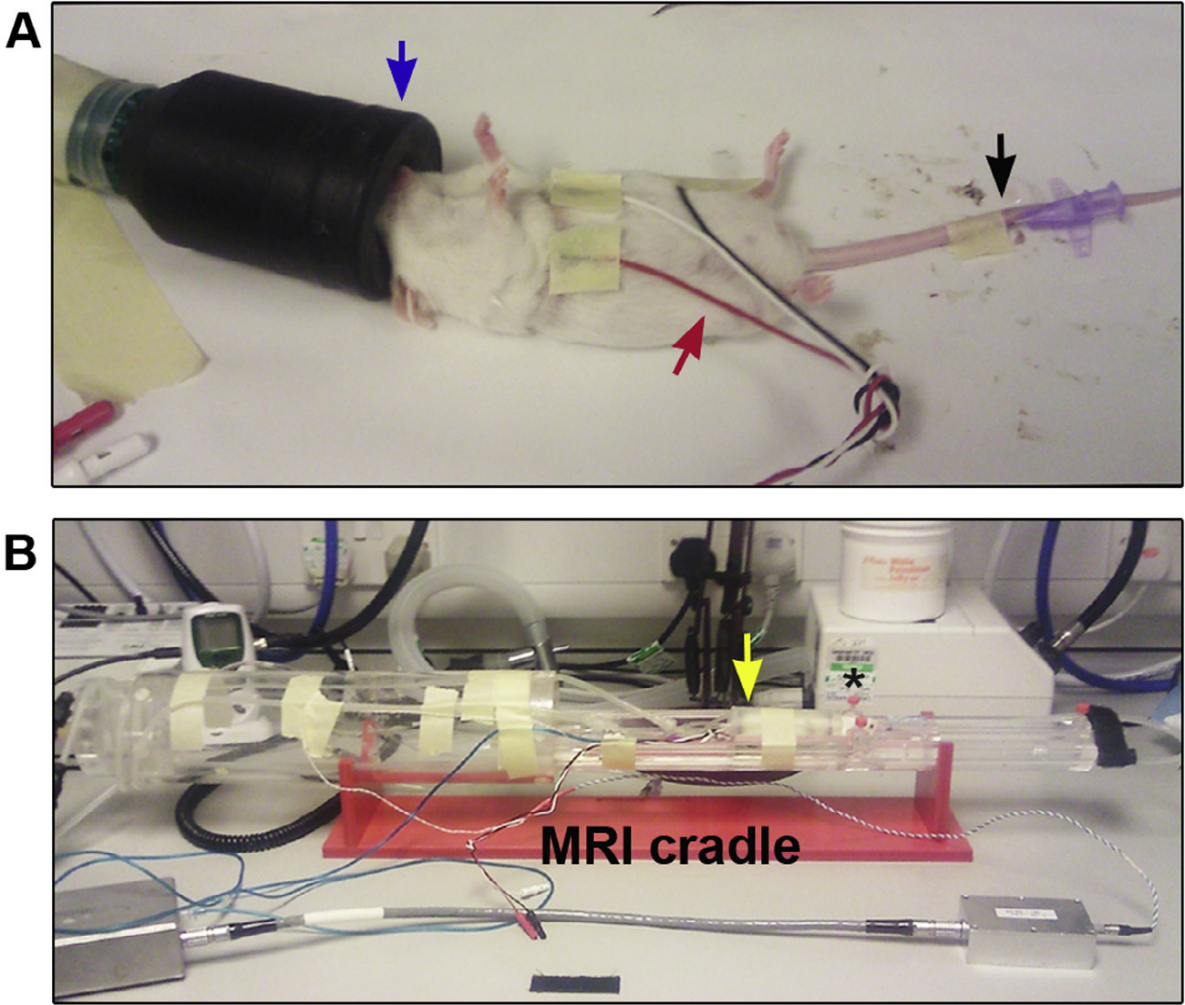
Preparation of mouse prior to contrast enhanced magnetic resonance imaging. A. The mouse was anaesthetised (blue arrow) and the tail vein cannulated (black arrow) to allow administration of contrast agent. Electrodes were placed on the thorax and abdomen to monitor respiration and heart rate (red arrow). B. The animal was the transferred into the MRI cradle and the head restrained with ear (*) and tooth bars to prevent movement during the scan. A rectal thermocouple was used to monitor body temperature (blue wire) which was maintained by an enclosed water circuit (yellow arrow) which surrounded the mouse. (For interpretation of the references to colour in this figure legend, the reader is referred to the web version of this article.)

MRI was performed at day 7, 14, 21 and 28 post-infection. Uninfected mice were also examined. All scans were performed as described previously [20]. Briefly, mice were anaesthetised with 1–2% isofluorane delivered in a 70:30 NO_2_:O_2_ mixture. The tail vein was cannulated with a 26 gauge × 19 mm cannula to facilitate contrast agent administration during MRI scanning (Fig.1A). The cannula was secured using super glue and masking tape and flushed with heparin to prevent the formation of blood clots. The animal was then placed prone into a mouse cradle and restrained using ear and tooth bars to minimise head movement. Anaesthesia was maintained throughout the procedures and respiration and heart rate were observed. Body temperature was continuously monitored via a rectal thermocouple and the animal maintained normothermic by an enclosed warm-water circuit (Fig.1B).

MRI was performed on a Bruker Biospec 7T/30 cm system equipped with an inserted gradient coil (121 mm ID, 400mT/m) and a 72 mm birdcage resonator. A surface coil was used for brain imaging. The scanning protocol consisted of a RARE T_1_ weighted scan [effective TE (echo time) 9 ms, TR (repetition time) 8000 ms, 20 averages, matrix 176 × 176, FOV (field of view) 17.6 × 17.6 mm, 20 contiguous coronal slices of 0.4 mm thickness). Following the RARE T_1_ weighted scan 0.1 mL of a solution containing 50 μL gadolinium-diethylenetriamine penta-acetic acid (Gd-DPTA Magnevist®; Bayer) and 50 μL of sterile water was injected via the tail vein cannula. Five minutes later the T_1_ weighted scan was repeated. Gd-DTPA cannot readily cross the intact blood brain barrier due to its charge and high molecular weight [24]. Extravasation of Gd-DTPA observed within the parenchyma demonstrates an impairment of the BBB integrity. A RARE T_2_ weighted scan (effective TE 76 ms, TR 5362 ms, 25 averages, matrix 176 × 176, FOV 17.6 × 17.6 mm, 20 contiguous coronal slices of 0.4 mm thickness) was then performed. On completion of the scan the animals were allowed to recover from anesthesia and the scanning procedure repeated at the next time-point.

Images were analysed using Image J software (http://rsbweb.nih.gov/ij/). Contrast enhancement maps were generated from the pre- and post-contrast T_1_ weighted scans according to the equation: *Enh = (S_post_* *– S_pre_)* *÷* *S_pre_* where *S_post_* = post contrast agent signal and *Spre* *=* pre-contrast agent signal. Regions of interest (ROIs) were manually defined to include the entire brain slice. Percentage signal change maps were generated by multiplying *Enh* by 100 and the mean percentage signal change for each brain slice calculated.

### 2.5 Statistical analysis

Data were analysed using analysis of variance methods in Minitab (Minitab Inc.). To identify significant differences between groups the General Linear Model (GLM) procedure, followed by *Tukey’s* multiple pair-wise comparison tests were applied. P values of less than 5% were considered statistically significant. Where appropriate data were log transformed prior to analysis. Group means were plotted to show means and their standard errors, and the size of treatment effects were estimated using differences between group means and their 95% confidence intervals.

## 3 Results

### 3.1 Histopathology

**Fig. 2:**
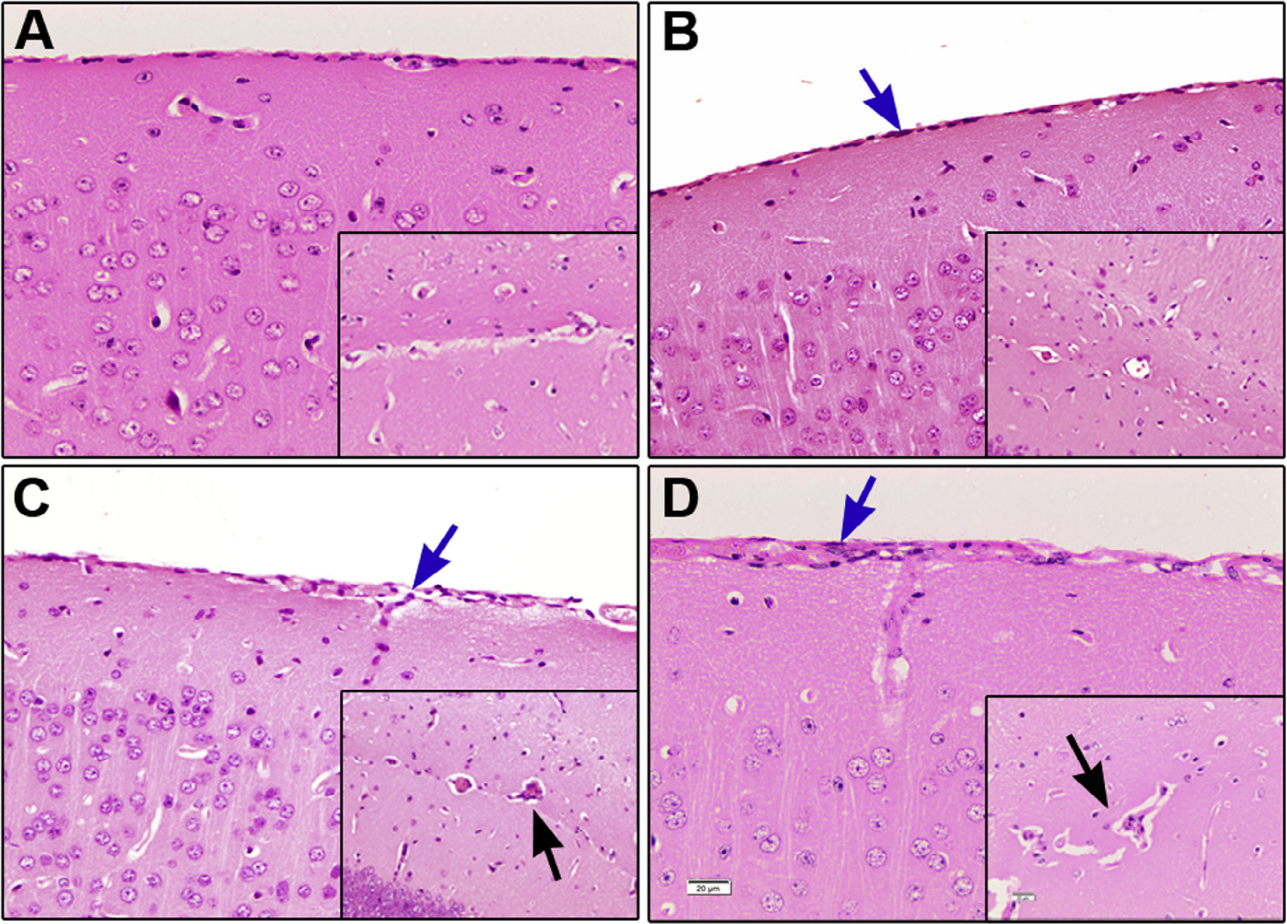
The neuroinflammatory reaction. Coronal brain sections, prepared at the level of the hippocampus, and stained with haematoxylin and eosin were employed to assess the neuroinflammatory reaction in uninfected mice (A) and mice infected with *T.b.brucei*. Inflammatory cells can be seen infiltrating the meninges (blue arrows) of the cerebral cortex on day 14 post-infection (B) and are most abundant at day 21 (C) and 28 (D) post-infection. Inserts show the development of mild perivascular cuffing of the vessels in the hippocampal fissure (black arrows) on days 21 (C) and 28 (D) post-infection. (magnification; ×200, calibration bar (20 µm) shown in panel D).

**Fig. 3:**
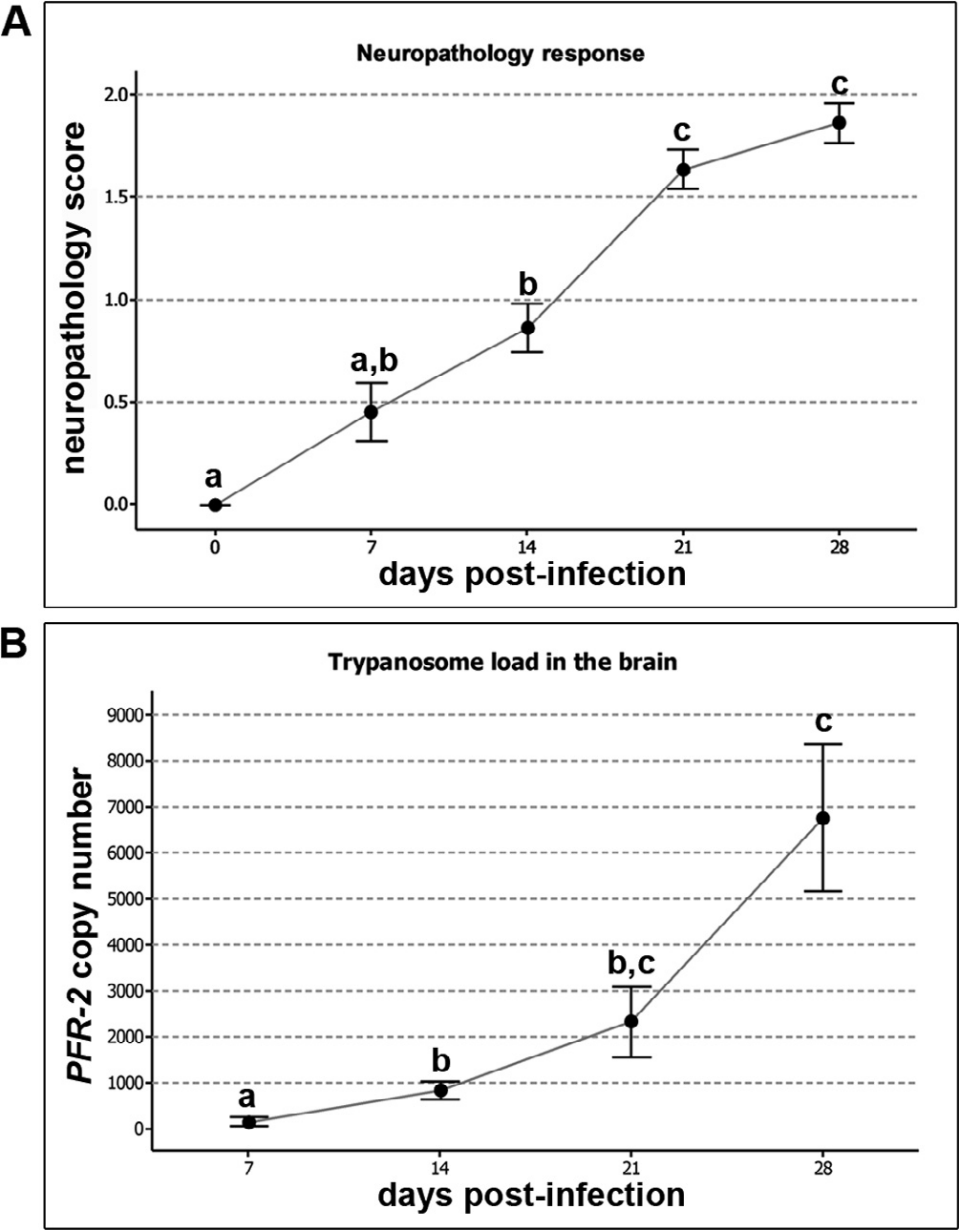
Assessing the neuroinflammatory response and brain parasite burden. A. The neuroinflammatory response scores were analysed in uninfected animals (n = 4) and animals killed at 7, 14, 21 and 28 days post-infection (n = 5 per group). An increase in the severity of the reaction can be seen as the infection progresses. B. The number of copies of the parasite specific *PFR-*2 gene in a 100 ng sample of DNA extracted from the brains animals killed at 7, 14, 21 and 28 days post-infection (n = 5 per group) was measured by Taqman PCR. The trypanosome burden within the CNS increases as the disease progresses with a significant increase in parasite load detected at 14 days post-infection compared to the earlier time-point. A and B. Dots represent the group mean, bars represent one standard error of the mean. The means of groups that do not share a letter are significantly different (p < 0.05) from each other.

Although a few inflammatory cells infiltrating the meninges were detected in some of the mice as early as day 7 after infection (Fig.2A) the mean neuropathology score [mean ± SE (0.400 ± 0.245)] for this group of mice was not significantly different [p = 0.382, 95% CI for the difference in mean score (95% CI) = −0.254, 1.054] to the uninfected group (0.00 ± 0.00). However, a progressive increase in the severity of the neuroinflammatory reaction was seen in the mice as the disease developed (Fig.3A) and by 14 days -post-infection the neuroinflammatory reaction, characterised by the presence of a mild meningitis (Fig.2B) (0.800 ± 0.122), was significantly (p = 0.012, 95% CI = 0.146, 1.454) higher than in uninfected mice (Fig.3A). A further significant increase (p < 0.05) in the severity of the neuropathological response was found in mice killed at days 21 (1.500 ± 0.158) and 28 (2.000 ± 0.000) post-infection compared to earlier time points (Fig.3A). In both of these groups the main features of the neuroinflammatory reaction were the presence of moderate inflammatory cell infiltration of the meninges and the perivascular space surround some of the blood vessels. The presence of perivascular cuffs was most often associated with the vessels situated in the hippocampal fissure (Fig.2C and D). Summary statistics for the neuroinflammatory score data are detailed in Supplementary Table 1.

### 3.2 Quantitative PCR

Trypanosome DNA was detected in the brains of animals killed at day 7 post-infection (155.5 ± 88.7) (Fig.3B). By day 14 post-infection the detectable parasite burden had increased significantly (841 ± 192; p = 0.005) compared to the earlier time-point (Fig.3B). The trypanosome load seen in the brains of animals killed at 21 days -post-infection showed a further rise (2332 ± 770). Although this was an increase of approximately 175% the burden was not significantly (p = 0.454) higher than that seen in animals on day 14 post-infection. A further rise in the number of parasites residing in the CNS was observed at day 28 post-infection (6766 ± 1607). The trypanosome load within the brain at this time-point was significantly higher than the levels detected at both 7 days -post-infection (p < 0.001) and14 days -post-infection (p = 0.010) but failed to reach significance when compared to animals killed on day 21 post-infection (p = 0.175). Summary statistics for the trypanosome load measured at each time-point are detailed in Supplementary Table 2.

### 3.3 Contrast-enhanced MRI

**Fig. 4:**
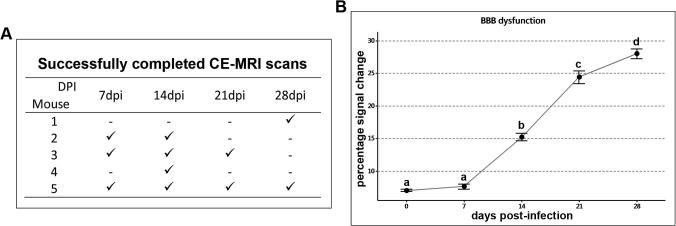
Assessing BBB dysfunction. A. Summary table indicating successful scans (✓) performed at each stage after infection. B. The degree of BBB dysfunction was determined by calculating the percentage signal change present in the brain following CE-MRI. Significant levels of barrier impairment were detected on day 14 post-infection compared with uninfected animals or those scanned at 7 days post-infection. A stepwise increase in BBB dysfunction was detected at days 21 and 28 post-infection. Dots represent the group mean (n = 2–4 in each group), bars represent one standard error of the mean. The means of groups that do not share a letter are significantly different (p < 0.05) from each other.

Successful scans were obtained from at least two animals at each time-point, details of completed scans are given in Fig.4A. It was not always possible to complete an MRI scan on each mouse at every time-point. Failures were encountered due to the technically demanding nature of the technique. In some instances cannulation of the tail vein proved impossible or the cannula became dislodged, while on other occasions the indwelling cannula became obstructed, most likely due to the presence of a blood clot, and prevented injection of the contrast agent.

**Fig. 5:**
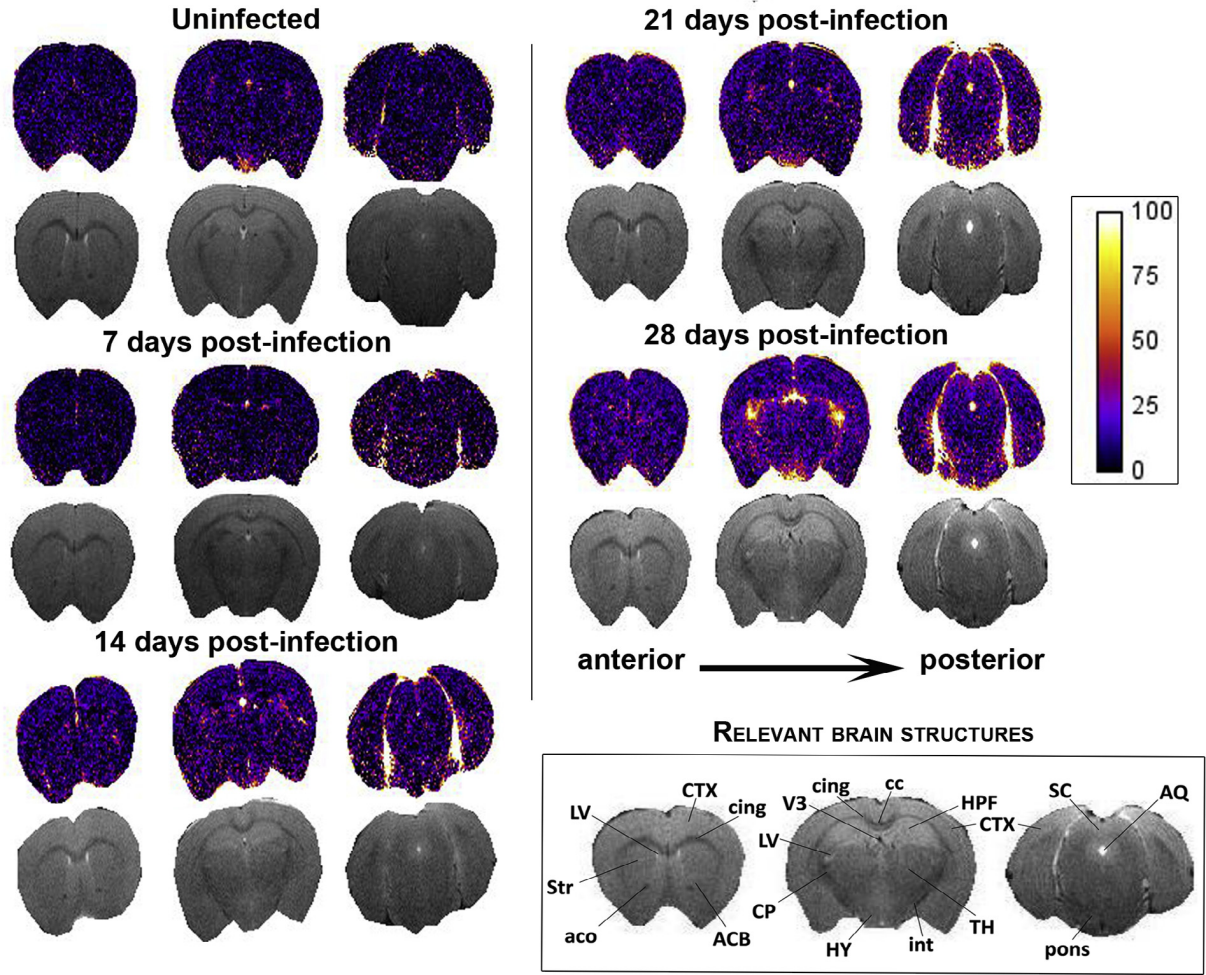
MRI percentage signal change maps. Percentage signal change maps (coloured), together with their respective T_2_ weighted scan images (grey), from an individual mouse, at each time-point studied are provided. In each case three slices, from the 20 measured in the procedure, are shown. The slices illustrated have been taken at similar levels in each mouse and cover the range of the scan. The colour bar represents the percentage signal change, with brighter colours corresponding to higher levels of change and therefore more severe BBB dysfunction. An increase in the intensity of the colours within the signal change maps can be clearly seen from day 14 post-infection and is most apparent in animals scanned on day 28 post-infection. Brain structures relevant to the images shown are described. LV, lateral ventricle; CTX, cerebral cortex; cing, cingulum; Str, striatum; aco,‘anterior commissure; ACB, nucleus accumbens; CP, Caudate putamen; V3, third ventricle; cc, corpus callosum; HPF, hippocampal formation; HY, hypothalamus; int, internal capsule; SC, superior colliculus; AQ, aqueduct (4th ventricle); pons, pons. (For interpretation of the references to colour in this figure legend, the reader is referred to the web version of this article.)

The base-line level of signal enhancement present in normal mice following administration of Gd-DPTA was determined in uninfected animals. The mean percentage signal change measured in these mice was 7.105 ± 0.162% (Figs. 4B and 5). A similar level of enhancement was seen in animals scanned at 7 days -post-infection (7.6 ± 0.397%). However by day 14 post-infection the mean percentage signal change (15.269 ± 0.586%) had risen significantly compared to uninfected mice (p < 0.001, CI = 5.21, 10.93) and mice scanned at 7 days -post-infection (p < 0.001, CI = 5.179, 9.992). At this point the most prominent areas of signal enhancement were found in the ventricular and hypothalamic areas although more subtle changes could be seen throughout the parenchyma (Fig. 5). A significant (p < 0.001, CI = 6.881, 11.476) increase in barrier dysfunction of approximately 9% when compared to 14 days -post-infection was detected on day 21 post-infection (24.447 ± 0.968%). A further stepwise rise was apparent in animals scanned on day 28 post-infection (28.056 ± 0.766%). The mean percentage signal change measured at day 28 post-infection was significantly higher (p < 0.005) than in all earlier time-points (Fig.4B). Although the ventricular and hypothalamic regions exhibited the highest level of contrast infiltration, increased signal change was also evident in the striatum, cerebral cortex, thalamus, and pons (Fig. 5). Diffuse BBB impairment was therefore present throughout the brain parenchyma and not confined to specific regions, such as the circumventricular organs and ventricles, where barrier function is intrinsically weaker. Summary statistics for the percentage signal enhancement data are detailed in Supplementary Table 3.

## 4 Discussion

The GVR 35 *T.b.brucei* mouse model is well established and characterised. In this model administration of trypanocidal drugs used to treat stage-1 infections, such as diminazene aceturate or suramin, will successfully cure the disease when they are given prior to day 21 post-infection. If chemotherapy is delayed beyond this point, only drugs tailored to cure stage-2 infections such as melarsoprol will prove effective [23]. Melarsoprol, unlike the stage-1 drugs, can cross the BBB to reach therapeutic concentrations within the brain. This information allows us to deduce that mice, infected with *T.b.brucei* GVR35, have entered stage-2 disease, with parasites established within the brain, by 21 days following initial infection [21], [25], [26]. The results of the present study indicate the development of statistically significant neuroinflammatory reactions, parasite burden and BBB dysfunction in animals at 14 days -post-infection, prior to the onset of established CNS-stage disease. All of the criteria investigated increased as the infection progressed through to the CNS-stage and demonstrated significantly greater levels on day 28 post-infection compared with day 14 post-infection.

The presence of trypanosomes situated in the brain parenchyma has been frequently documented in rodent models of HAT during both the chronic [16], [17], [27] and more acute stages of the infection [22], [28]. Furthermore, manipulation of the immunological microenvironment has been shown to alter the ability of the parasites to enter the neuropil and mice deficient in CXCL10 or IFN-γ show reduced parasite numbers in the parenchyma or fail to develop CNS-stage infections [29], [30]. In this study, Taqman PCR was used to assess the number of trypanosome present within the brain. This technique is highly sensitive and showed that parasite DNA was present at low levels as early as 7-days after infection and found in rising quantities at 14, 21 and 28 days -post-infection. Taqman PCR does not provide details regarding the location of the parasites within the brain tissue. It is possible that small quantities of blood, remaining in the blood-vessels following perfusion, could be responsible for the low numbers of trypanosomes identified during the early stage of the infection. At later time-points a more substantial parasite burden was detected. However, since we have no information regarding the location of the parasites, it could be argued that they may not be present in the brain parenchyma but situated in the meninges, ventricles or other areas such as the circumventricular organs where the blood-vessel epithelium is fenestrated. In fact, recent investigations in a rat model of infection, using freeze fracture electron microscopy, suggested that trypanosomes do not reside in the neuropil of the brain, until the terminal stages of the infection, but persist between the cell layers in the *pia mater* where the population size is controlled by prostaglandin-D_2_ [31]. The increasing brain parasite burden detected here argues against this scenario in this study; and since no cyclical fluctuations in the trypanosome population were apparent this would suggest that the parasites were not situated in the choroid plexus, or CSF [31], [32]. The presence of high levels of residual DNA from dead or dying parasites also seems unlikely due to the high turnover of CSF in the brain [33], [34], [35].

The apparent inception of neurological involvement during early-stage infections has been identified previously in animal models of trypanosomiasis. The presence of trypanosomes within the CNS, only hours following infection, was demonstrated in a murine model of sleeping sickness [28]. Here the authors used intravital microscopy to show fluorescently tagged *T.b.rhodesiense* IL1852 and *T.b.brucei* GVR35 in the cortical microvasculature and brain parenchyma 5 h following intravenous infection. A recent study by Laperchia et al. [22] demonstrated the presence of parasites and T-cells in the brain parenchyma at day 9 post-infection in a *T.b.brucei* rat model of HAT. The authors present confocal images illustrating parasite traversal of the blood vessel endothelia to enter the neuropil on day 9 post-infection and show high parasite burdens by day 21 post-infection. In addition, this study detected the occurrence of sleep-onset rapid eye movement (SOREM) episodes in the rats during the first week following infection and demonstrated that the number and duration of these episodes did not correlate with the trypanosome load found in the brain parenchyma. SOREM periods represent a disruption of the normal sleep architecture and are typically thought to be associated with CNS-stage infections [36]. However, in the Laperchia study they became apparent early after infection demonstrating the presence of neurological features associated with early-stage disease. In human cases of sleeping sickness, caused by *T.b.rhodesiense*, a similar disconnect between the incidence of neurological signs, and development of stage-2 infection occurs [37]. Patients presenting with either early and late-stage infections exhibited symptoms such as altered gait, tremors, incontinence, cranial nerve palsy, somnolence and reduced Glasgow coma score. Indicating that these neurological signs can occur prior to progression to stage-2 disease.

The few reports detailing the neuropathological features in post-mortem samples from fatal cases of HAT describe the development of a diffuse meningoencephalitis, with the inflammatory infiltrate comprised of macrophages, lymphocytes and plasma cells [5], [6], [38]. Astrocyte and microglial cell activation was also noted. The neuroinflammatory changes were most apparent in the white matter of the cerebral hemispheres but also occurred in the circumventricular areas. The neuroinflammatory picture has also been described in rodent [9], [16], [27], [29], [39], [40], [41], [42], [43] and primate [7], [44], [45], [46] models of trypanosome infection. Although these studies reported a similar response to that described in human cases the development of a meningoencephalitis, with inflammatory cells infiltrating the brain parenchyma, was only seen on a few occasions and was more commonly associated with treatment failures [7], [45], [47]. In the *T.b.brucei* murine model used in this study only mild to moderate neuroinflammation, with inflammatory cells present in the meninges and infiltrating the perivascular space in the later stages of the infection, was found corresponding with the reaction previously reported in the alternative animal models.

Contrast-enhanced MRI has been used previously to demonstrate BBB dysfunction during stage-2 infections [20]. In the present study these findings have been confirmed and extended to examine earlier time-points following infection. It is now clear that barrier impairment becomes apparent during the haemolymphatic stage of the infection since a significant enhancement in signal intensity, to a mean of 15.85%, was apparent on day 14 post-infection compared to uninfected animals and those examined at the earlier time-point. The degree of barrier damage was further augmented to 25.41% at 21 days -post-infection and 28.82% on day 28 post-infection indicating that the trypanosome infection results in a progressive loss of BBB function. Although the increased signal intensity was most marked in the ventricular regions, infiltration of the contrast agent was not confined to these areas (Fig. 5) but found throughout the brain slice. Since signal enhancement was present throughout the brain parenchyma it is unlikely that the influx of contrast agent is restricted to traversal of the blood-CSF barrier and seems also to occur through the BBB. In earlier studies using an *in situ* perfusion model, the authors found evidence of BBB impairment, assessed by measuring [^14^C]sucrose concentrations in the parenchyma, during the late-stage of infection in a BALB/c mouse model [18], [48]. Philip et al. [17] also found evidence of BBB dysfunction in a rat model of HAT. In this case rhodamine dye was injected into the jugular vein at specific time-points following infection. Low levels of dye leakage into the brain, at first confined to the thalamus and hypothalamus, were seen at 21 days post-infection with widespread penetration of both the white and grey matter evident by day 40 after infection. Again, this study fails to identify any barrier impairment at the earlier time-points after infection. The disparity between the current study and those previously published is likely a result of the differing methodologies used. MRI uses contemporary contrast agents that result in low levels of signal enhancement in the brain even in normal animals whereas rhodamine may remain excluded until more overt changes in barrier function develop. A similar situation exists with the use of sucrose as a marker of BBB permeability since this compound is considered to be excluded from the brain parenchyma under normal conditions [49]. Interestingly, BBB dysfunction, assessed using the CSF/serum quotient of albumin, were detected in 6% of patients presenting with stage-1 *T.b.rhodesiense* infections [37]. This figure increased to 42% when the stage-2 cases were examined indicating the development of progressive BBB impairment with advancing disease.

This results of this study highlight the apparent inconsistency between the trypanosome burden in the brain and the degree of BBB impairment as evidenced by the severity of the neuroinflammatory reaction present within the brain of trypanosome infected animals. Although statistical differences between the neuropathology scores were apparent between the groups of mice examined through the various stages of disease progression the neuroinflammatory response remained mild to moderate throughout with no inflammatory cells infiltrating the neuropil. This suggests that the mechanisms in place within the brain to control the inflammatory response and maintain homeostasis remain largely effective despite the barrier dysfunction and the increasing parasite burden. Furthermore, the neuroanatomical expression patterns of tight junction proteins and adhesion molecules differ between the BBB, the blood-meningeal barrier and the blood-CSF barrier [50]. This could result in differential infiltration of specific regions dependent on the particular immune environment. Several host and parasite factors have been implicated as key determinants of disease progression. These include molecules regulating both the innate and adaptive immune response. For example, Toll-like receptor (TLR) 2- and TLR9- MyD88 signalling have been shown to stimulate the expression of inflammatory mediators such as TNF-α and IFN α/β which initiate leucocyte and parasite transmigration into the CNS [51] and additional inflammatory mediators such as IFN-γ [29], [51] and CXCL10 [51] have also been shown to play a vital role in this process. Furthermore, the migration of leucocytes across the BBB as well as the laminin subtype composition of the basement membrane, influence the ability of trypanosomes to enter the CNS [29], [42] and increased levels of ICAM and E-selectin have been demonstrated following infection [42]. Other non-immune molecules such as matrix metalloproteases [42], and parasite cysteine proteases [52] also appear to affect BBB penetration by the parasites.

The ability to stage human infection accurately has become a focus of attention in recent years. The current WHO guidelines suggest that the presence of more than five white blood cells/µL of CSF or the presence of trypanosomes indicates that the infection has entered stage-2 [1]. Nevertheless, WHO recognise that many of the patients presenting with between six and 20 white blood cells/µL are likely to be in an intermediate stage, prior to the onset of stage-2 disease [1]. Precise disease staging is pivotal in determining the correct chemotherapeutic approach for optimal therapy. Treatment of stage-2 infections with suramin or pentamidine will fail to cure the infection resulting in relapse or exacerbation of the neuroinflammatory reaction while unnecessary use of toxic stage-2 drugs, such as melarsoprol, should be avoided due to the high incidence of extremely severe adverse reactions [4], [53]. The results reported here show that BBB dysfunction and neuroinflammation manifest before the onset of stage-2 infections. In addition, parasite DNA can be detected in the brain tissue early after infection. These findings challenge the prevailing notion that parasites simply cross the BBB to enter the CNS and initiate inflammation during the transition from acute to CNS-stage disease, and also highlight the need for further research into the neuropathogenesis of trypanosomiasis. In addition the results reported here question the value of the criteria currently employed to defining stage-1 and stage-2 HAT and highlight a requirement to elucidate accurate markers of stage-2 infection to facilitate the development of improved, accurate diseases staging tools.
