# Supplementary material for: Delineating neuroinflammation, parasite CNS invasion, and blood-brain barrier dysfunction in an experimental murine model of human African trypanosomiasis
Source: Methods. 2017 Aug 15;127:79–87. doi: 10.1016/j.ymeth.2017.06.015 (PMC5595161; doi:10.1016/j.ymeth.2017.06.015)
Supplement: Supplementary data [file mmc1.docx]

|  | 0 days post-infection | 7 days post-infection | 14 days post-infection | 21 days post-infection | 28 days post-infection |
| --- | --- | --- | --- | --- | --- |
| 7 days post-infection | p=0.382  (-254, 1.054) |  |  |  |  |
| 14 days post-infection | p=0.012  (0.146, 1.454) | p=0.327  (-0.217, 1.017) |  |  |  |
| 21 days post-infection | p<0.001  (0.846, 2.154) | p<0.001  (0.483, 1.717) | p=0.022  (0.083, 1.317) |  |  |
| 28 days post-infection | p<0.001  (1.346, 2.654) | p<0.001  (0.983, 2.217) | p<0.001  0.583, 1.817) | p=0.148  (-0.117, 1.117) |  |
| Mean + SE  number | 0.000 + 0.000  4 | 0.400 + 0.245  5 | 0.800 + 0.122  5 | 1.500 + 0.158  5 | 2.000 + 0.000  5 |

**Supplementary table 1.** **Neuropathological reaction.** Mice were infected with *T. b. brucei* and killed at 0, 7, 14, 21 and 28 days post-infection. The severity of the neuroinflammatory reaction was graded on a scale of 0-4 where 0 is normal and 4 shows a severe meningoencephalitis. The figures in the body of the table demonstrate the comparisons, in terms of statistical significance, between the time points shown in the row and column headings for each time-point studied. The *p-*values and 95% confidence intervals are based on ANOVA (GLM) followed by Tukey’s *post-hoc* analysis. The mean severity score + the standard error and the number of mice per group are also shown.

|  | 7 days post-infection | 14 days post-infection | 21 days post-infection | 28 days post-infection |
| --- | --- | --- | --- | --- |
| 14 days post-infection | p=0.0053  (0.620, 3.798) |  |  |  |
| 21 days post-infection | P=0.0003  (1.458, 4.636) | p=0.4545  (-0.751, 2.427) |  |  |
| 28 days post-infection | p<0.0001  (2.661, 5.839) | p=0.0099  (0.452, 3.630) | p=0.1746  (-0.386, 2.792) |  |
| Mean + SE  number | 155.5 + 88.7  5 | 841 + 192  5 | 2332 + 770  5 | 6766 + 1607  5 |

**Supplementary table 2.** **Trypanosome CNS burden.** Mice were infected with *T. b. brucei* and killed at 0, 7, 14, 21 and 28 days post-infection. The trypanosomes load in the brain was assessed using Taqman PCR designed to amplify the trypanosome specific *PFR*2 gene. The figures in the body of the table demonstrate the comparisons, in terms of statistical significance, between the time points shown in the row and column headings for each time-point studied. The *p-*values and 95% confidence intervals are based on ANOVA (GLM) followed by Tukey’s *post-hoc* analysis performed following a logarithmic transformation [log+1]. The mean *PFR*2 copy number + the standard error and the number of mice per group are also shown.

|  | 0 days post-infection | 7 days post-infection | 14 days post-infection | 21 days post-infection | 28 days post-infection |
| --- | --- | --- | --- | --- | --- |
| 7 days post-infection | p=0.950  (-1.588, 2.744) |  |  |  |  |
| 14 days post-infection | p<0.001  (6.137, 10.189) | p<0.001  (5.559, 9.611) |  |  |  |
| 21 days post-infection | p<0.001  (14.920, 19.764) | p<0.001  (14.342, 19.186) | p<0.001  (6.881, 11.476) |  |  |
| 28 days post-infection | p<0.001  (18.528, 23.372) | p<0.001  (17.950, 22.794) | p<0.001  (10.489, 15.084) | p=0.002  (0.955, 6.261) |  |
| Mean + SE  number | 7.105 + 0.162  3 | 7.683 + 0.397  3 | 15.269 + 0.586  4 | 24.447 + 0.968  2 | 28.056 + 0.766  2 |

**Supplementary table 3.** **Contrast enhanced -MRI.** Mice were infected with *T. b. brucei* and scanned at 0, 7, 14, 21 and 28 days post-infection. The degree of BBB dysfunction was calculated as the percentage signal change found following administration of Gd-DPTA contrast agent. The figures in the body of the table demonstrate the comparisons, in terms of statistical significance, between the time points shown in the row and column headings for each time-point studied. The *p-*values and 95% confidence intervals are based on ANOVA (GLM) followed by Tukey’s *post-hoc* analysis. The mean percentage signal change + the standard error and the number of mice per group are also shown.
